# *Starmerella bombicola* and *Saccharomyces cerevisiae* in Wine Sequential Fermentation in Aeration Condition: Evaluation of Ethanol Reduction and Analytical Profile

**DOI:** 10.3390/foods10051047

**Published:** 2021-05-11

**Authors:** Laura Canonico, Edoardo Galli, Alice Agarbati, Francesca Comitini, Maurizio Ciani

**Affiliations:** Dipartimento Scienze della Vita e dell’Ambiente, Università Politecnica delle Marche, Via Brecce Bianche, 60131 Ancona, Italy; l.canonico@univpm.it (L.C.); e.galli@pm.univpm.it (E.G.); a.agarbati@pm.univpm.it (A.A.); f.comitini@univpm.it (F.C.)

**Keywords:** ethanol reduction, *Starmerella bombicola*, oxygen, wine, analytical profile

## Abstract

In the last few decades, the increase of ethanol in wine, due to global climate change and consumers’ choice is one of the main concerns in winemaking. One of the most promising approaches in reducing the ethanol content in wine is the use of non-*Saccharomyces* yeasts in co-fermentation or sequential fermentation with *Saccharomyces cerevisiae*. In this work, we evaluate the use of *Starmerella bombicola* and *S. cerevisiae* in sequential fermentation under aeration condition with the aim of reducing the ethanol content with valuable analytical profile. After a preliminary screening in synthetic grape juice, bench-top fermentation trials were conducted in natural grape juice by evaluating the aeration condition (20 mL/L/min during the first 72 h) on ethanol reduction and on the analytical profile of wines. The results showed that *S. bombicola*/*S. cerevisiae* sequential fermentation under aeration condition determined an ethanol reduction of 1.46% (*v*/*v*) compared with *S. cerevisiae* pure fermentation. Aeration condition did not negatively affect the analytical profile of sequential fermentation *S. bombicola*/*S. cerevisiae* particularly an overproduction of volatile acidity and ethyl acetate. On the other hand, these conditions strongly improved the production of glycerol and succinic acid that positively affect the structure and body of wine.

## 1. Introduction

Ethanol is the main product in wine produced by yeast during alcoholic fermentation. During the last two decades, in many different geographical areas, the average alcohol level has risen about 2% (*v*/*v*) [1].

Generally, the alcohol level in wine is between 12 and 14% (*v*/*v*) with some exception the different varieties of wines [2].

The climatic changes recorded in recent years have resulted in grapes with high sugar concentrations, which is reflected in wines with increased ethanol content. Wines with high ethanol content are associated with health issues, economic and quality aspects [3,4,5,6,7,8,9,10,11,12].

Indeed, high alcohol levels in wine compromise the organoleptic properties of the product increasing the hotness and viscosity, and decreasing sweetness, intensity, and aromatic flavors [13,14,15,16,17,18,19,20]. The combination of these aspects (organoleptic, economic and health issues) in wine with high ethanol content has led to the development of technological strategies to produce wines with a reduced alcohol level without affecting flavour profile and sensorial characteristics [21]. For these reasons, many strategies in reduce ethanol content in wine during the winemaking process have been proposed, such as viticultural, pre-fermentation, fermentation and post fermentation practices [1,8,22,23].

A suitable strategy for reducing the alcohol level of wine is the use of non-*Saccharomyces* yeasts able to use different pathways for sugar convert (respiration, alcoholic fermentation, and glycerol-pyruvic metabolism) [24,25,26]. Biotechnological processes under different fermentation conditions with non-*Saccharomyces* in co-culture or sequential fermentation with *S. cerevisiae* starter strain were proposed [22,27,28,29,30,31,32].

In sequential inoculation, the non-*Saccharomyces* yeast strain is inoculated in the grape juice in the first stage of fermentation (48–72 h). This procedure allows the non-*Saccharomyces* strain to take advantage favouring its metabolic activity. In particular, the non-*Saccharomyces* yeasts could affect the wines by producing a low ethanol yield, low volatile acidity and/or enhancement of specific analytical and aromatic compounds [33,34,35,36]. Different researches showed that the physiological features of *Metschnikowia pulcherrima Lachancea thermotolerans,* *Torulaspora delbrueckii, Starmerella* and *Zygosaccharomyces* spp. strains are suitable for lower ethanol content in wine in the presence of oxygen. According with the results obtained by controlled aeration fermentations the ethanol reduction was for *M. pulcherrima* 1.6% (*v/v)*, *T. delbrueckii* 0.9% (*v*/*v*), *Z. bailii* 1.0% (*v*/*v*), and *S. bacillaris* 0.7% (*v*/*v*) compared with *S. cerevisiae* wine [31,37]. In recent previous work, *Starmerella bombicola* (formerly *Candida stellata*) was evaluated for ethanol reduction in wine in a static condition and in a immobilized form with promising results [32]. However, immobilized cells are a modality of inoculum, quite complex and difficult to apply under an industrial vinification condition.

Previously, a strain of *S. bombicola* was proposed to enhance the glycerol content of wine in immobilized form to overcome its low fermentation rate [38]. Indeed, the general enological traits of *S. bombicola* strains showed low fermentation rate and low fermentation power (4–5% vol. of ethanol) together with some interesting positive features as high glycerol and succinic acid production.

In the present work, with the aim to reduce the ethanol content in wine, *S. bombicola*/*S. cerevisiae* sequential fermentations were evaluated under partial aeration condition. The analytical composition and aromatic profile of the final wines were also evaluated.

## 2. Materials and Methods

### 2.1. Yeast Strains

The non-*Saccharomyces* yeast strain used in this study was *S. bombicola* DiSVA 66 (formerly named *Candida stellata* DBVPG 3827; Industrial Yeast Collection of the University of Perugia) and evaluated in a previous work in immobilized form [32]. *S. cerevisiae* commercial strain Lalvin EC1118 (Lallemand Inc., Toulouse, France) was used in pure (control) and sequential fermentation trials. These strains were maintained on YPD agar medium (1% yeast extract, 2% peptone, 2% D-glucose, and 1.8% agar) at 25 °C for 48 h, and stored at 4 °C.

### 2.2. Preliminary Screening on Synthetic Grape Juice (SGJ)

Modified YPD medium (0.5% yeast extract, 0.1% peptone, 2% dextrose, all *w*/*v*) was used to obtain biomass for fermentation trials. *S. bombicola* cells were incubated at 25 °C for 72 h in a rotary shaker (150 rpm). This biomass was harvested by centrifugation, washed three times with sterile distilled water.

To optimize the cell concentration of *S. bombicola,* screening was conducted on SGJ, and prepared according to the protocol reported by Ciani and Ferraro [38]. The trials were carried out in 100 mL flask containing 70 mL SGJ under static and agitation condition (200 rpm rotary shaker) at 22 ± 1 °C in triplicate. The inoculum of *S. bombicola* was 1 × 10^8^ cells/mL and 5 × 10^7^ cells/mL followed three days, by *S. cerevisiae* (1 × 10^6^ cells/mL). Ethanol content, volatile acidity and glycerol content were analyzed. The fermentation trial, which showed the best reduction in alcohol content was selected to set up fermentation in Natural Grape Juice (NGJ).

### 2.3. Natural Grape Juice (NGJ) Fermentation Trials

Natural grape juice (NGJ) (Verdicchio white grape variety), used for trials, showed the following characteristics: pH, 3.32; total acidity, 5.17 g/L; free SO_2_, 9 mg/L; total SO_2_, 18 mg/L; malic acid, 3.1 g/L; initial sugar content, 218 g/L; yeast assimilable nitrogen (YAN) 121 mg N/L.

The 2-L Bench-top bioreactor (Biostat^®^ B; B. *Braun* Biotech Int., Goettingen, Germany) containing 1.5 L of natural grape juice under gentle agitation (60 rpm/min) was used for fermentation trials. The temperature was 22 °C with an inoculation level of 5 × 10^7^ cells/mL of *S. bombicola* obtained using the procedure described above. Aerobic condition was maintained using 20 mL/L/min of air flow during the initial 72 h, while in semi-anaerobic condition no aeration was applied. In sequential fermentations, *S. cerevisiae* was inoculated after 72 h (1 × 10^6^ cells/mL). Pure fermentations of *S. cerevisiae* (inoculum 1 × 10^6^ cells/mL) were used as controls under gentle agitation (60 rpm/min, semi-anaerobic condition).

A specific enzymatic kit (Megazyme International Wicklow Ireland) was used to evaluate the sugar consumption during the fermentation to monitor fermentation kinetics. Biomass evolution was evaluated by viable cell count (CFU/mL) on Lysine Agar selective medium and WL nutrient agar (Oxoid, Hampshire, UK) [39]. Wild non-*Saccharomyces* yeasts (WNS) were easily distinguished by *S. bombicola* through a macro- and microscopic characterization of colony on WL nutrient agar. The fermentations were carried out in triplicate.

### 2.4. Analytical Procedures

Total acidity, volatile acidity, pH and ethanol content were determined according to the Official European Union Methods [40]. The final samples, prepared following the procedure of Canonico et al. [41], were directly injected into a gas chromatography system (GC-2014; Shimadzu, Kjoto, Japan) to quantify acetaldehyde, ethyl acetate, n-propanol, isobutanol, amyl and isoamyl alcohols. Solid-phase microextraction (HS-SPME) method with the fiber. Divinylbenzene/Carboxen/Polydimethylsiloxane (DVB/CAR/PDMS) (Sigma-Aldrich, St. Louis, MO, USA) was used to determine the main volatile compounds desorbed by inserting the fiber into gas chromatograph GC (GC-2014; Shimadzu, Kjoto, Japan) The compounds were identified and quantified using external calibration curves [42].

Glucose and fructose (K-FRUGL), glycerol (K-GCROL) and succinic acid (K-SUCC) were analyzed using specific enzyme kits (Megazyme International, Wicklow Ireland).

### 2.5. Statistical Analysis

Experimental data for the main analytical characters of wine have been subjected to analysis of variance (ANOVA) using the statistical software package JMP^®^ 11. Duncan test was used to determine the significant differences (*p*-values were <0.05).

## 3. Results

### 3.1. Preliminary Screening on Synthetic Grape Juice

**Figure 1:**
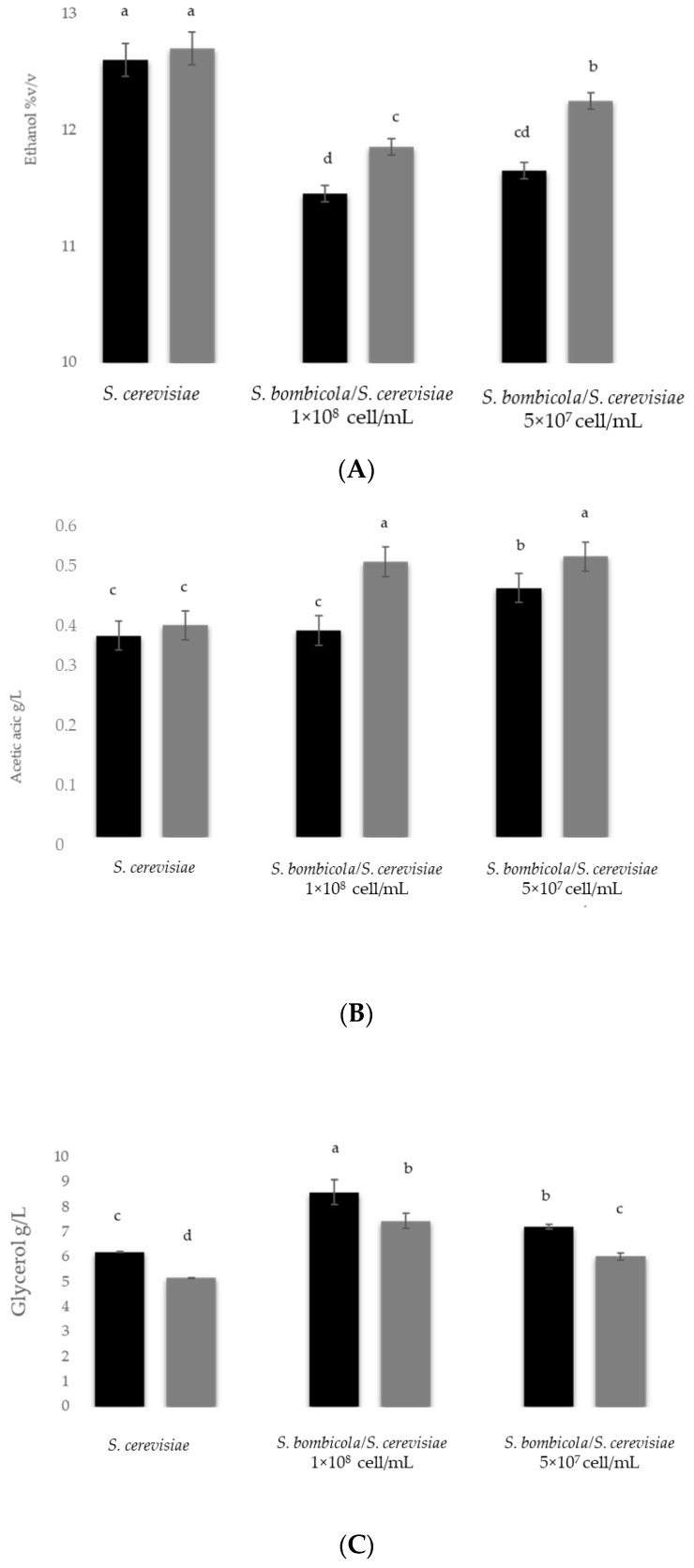
Ethanol content, (**A**) volatile acidity, (**B**) and glycerol content (**C**) of preliminary screening in sequential fermentation in static and agitation condition in *Synthetic Grape Juice*. 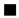 Agitation condition; 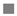 static condition. Data with different superscript letters (^a,b,c,d^) are different according to Duncan tests (0.05%).

The results of the ethanol content of screening trials, carried out under static and agitation conditions, was reported in Figure 1A.

*S. bombicola*/*S. cerevisiae* sequential fermentation in agitation condition significant enhanced the ethanol reduction if compared with static one and *S. cerevisiae* pure culture both in static and agitation condition. In particular, *S. bombicola*/*S. cerevisiae* sequential fermentation 10^8^ cells/mL and 5 × 10^7^ cells/mL achieved an ethanol reduction of 1.25% (*v*/*v*), and 1.05% (*v*/*v*), respectively in comparison with *S. cerevisiae* pure culture (in both conditions). Moreover, the ethanol content in the trials with inoculum level 5 × 10^7^ cells/mL in agitation condition was comparable with that obtained with 10^8^ cells/mL.

In relation to the volatile acidity (Figure 1B), the fermentation trials at different inoculum level of *S. bombicola* showed in general similar values exhibited by *S. cerevisiae*. A significant increase was detected only with *S. bombicola*/*S. cerevisiae* sequential fermentation in static condition using different inoculation levels (0.53 g/L acetic acid). The aeration conditions determined a general enhancement of glycerol production in all fermentation trials. In particular, a significant increase was showed in *S. bombicola*/*S. cerevisiae* 1 × 10^8^ cells/mL sequential fermentations (8.58 g/L) compared with *S. cerevisiae* pure culture with the exception of *S. bombicola*/*S. cerevisiae* 5 × 10^7^ cells/mL in static condition (Figure 1C).

Considering the similar results obtained and the most practice application in vinery condition of the lower inoculum level, *S. bombicola* at inoculum level 5 × 10^7^ cell/mL was identified for the further bench-top fermentation trials in NGJ. Using the following fermentation conditions: Semi-anaerobic (gently agitation 60 rpm) and aeration flow of 20 mL/L/min during the first 72 h.

### 3.2. Bench-Top Fermentation Trials

#### 3.2.1. Biomass Evolution and Sugar Consumption in Natural Grape Juice (NGJ)

**Figure 2:**
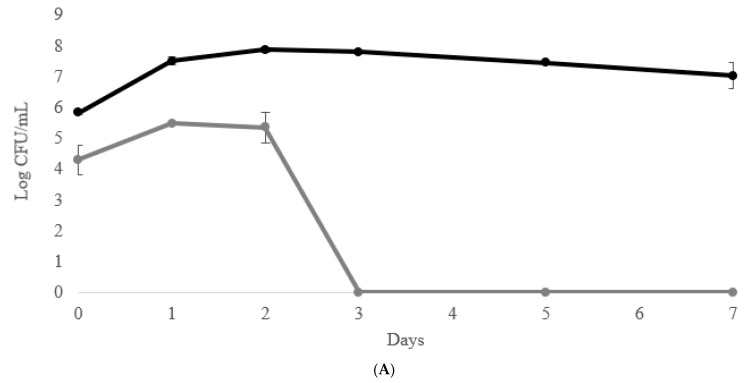

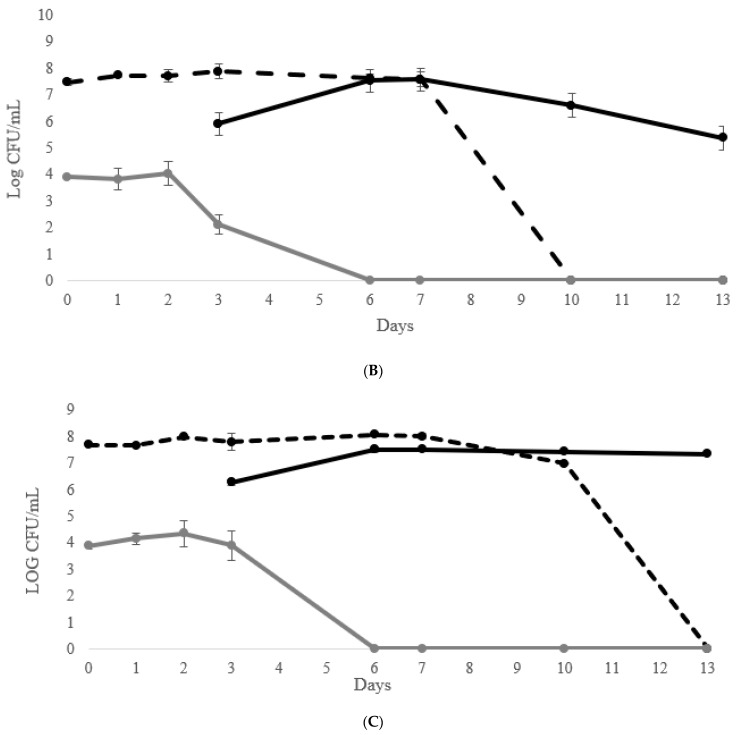
Growth kinetics in sequential fermentation trials *S. bombicola*/*S. cerevisiae* and control *S. cerevisiae* on natural grape juice (NGJ). (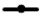) *S. cerevisiae*; (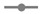) Wild non-*Saccharomyces*; (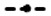) *S. bombicola*. (**A**) control-pure fermentation with *S. cerevisiae* inoculum; (**B**) semi-aerobic condition (no aeration); (**C**) with aeration (20 mL/L min of air flux during the first 72 h).

The growth kinetics are reported in Figure 2. The pure *S. cerevisiae* fermentation (Figure 2A) achieved the maximum cell concentration (c.a. 10^8^ cells/mL) in 2 days maintaining this cell concentration until the end of the fermentation. When *S. cerevisiae* reached the maximum cell concentration, the wild non-*Saccharomyces* yeasts (WNS) present in the natural grape juice, decreased until disappeared.

A similar trend in biomass evolution of *S. cerevisiae* and WNS was shown in semi-anaerobic conditions (Figure 2B). In terms of the *S. bombicola* population, the differences between semi-anaerobic and air flow addition were shown. The sequential fermentations carried out with air flow (20 mL/L/min of air flux during the first 72 h) (Figure 2C) maintained high level (>10^7^ cfu/mL) until the tenth day, while in semi-aerobic condition high biomass concentration where maintained only until seventh day (Figure 2B). Thus, *S. bombicola* fermentation trials with air flow showed a higher persistence of viable cells in comparison with sequential fermentation in semi-anaerobic condition. In relation to the evolution of WNS, *S. cerevisiae* pure culture showed a strong control of WNS that disappeared on the third day of fermentation, while WNS with *S. bombicola*/*S. cerevisiae* sequential fermentation disappeared at sixth day of fermentation (in both conditions: with and without air flux).

The duration of fermentation process was approximately 13 days for both the sequential fermentations while *S. cerevisiae* pure culture, as expected, completed the fermentation in 7 days.

**Figure 3:**
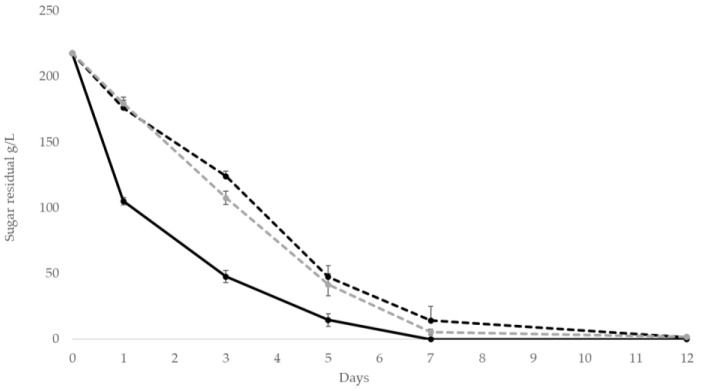
Sugar consumption kinetics in sequential fermentation trials *S. bombicola*/*S. cerevisiae* and control *S. cerevisiae* on natural grape juice (NGJ) in static and aeration condition. (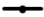) *S. cerevisiae*; (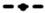) *S. bombicola*/*S. cerevisiae* static and (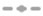) *S. bombicola*/*S. cerevisiae* 20 mL/L/min oxygen.

The sugar consumption (Figure 3) confirmed the fermentation trend: the sequential fermentations showed a comparable trend *S. cerevisiae* pure culture exhibited a faster fermentation kinetics with a half of the sugar consumed after 24 h of fermentation.

#### 3.2.2. Main Fermentation Parameters in Natural Grape Juice (NGJ)

The main fermentation parameters determined at the end of fermentation are reported in Table 1.

In relation to the ethanol content, in comparison with *S. cerevisiae* pure culture, *S. bombicola*/*S.cerevisiae* air flow exhibited an ethanol reduction of 1.46% (*v*/*v*). Whereas, *S. bombicola*/*S. cerevisiae* static condition led an ethanol reduction of 0.21% (*v*/*v*). This trend was reflected by the ethanol yield that was significant significantly lower in *S. bombicola*/*S. cerevisiae* with air flow in comparison with other fermentation trials. While, volatile acidity amounts were comparable among the fermentations, sequential fermentation led a general increase in final glycerol content. In particular, *S. bombicola* sequential fermentation air flow supplied showed a significant increase in this compound (more than 3-fold of *S. cerevisiae*). However, the results also exhibited an increase in glycerol content in static sequential fermentations, indicating the effect of *S. bombicola* in glycerol production. Aeration condition also determined a significant increase in succinic acid.

#### 3.2.3. The Main Volatile Compounds in Natural Grape Juice

The data of the main volatile compounds are reported in Table 2.

In relation to the higher alcohols, the sequential fermentation trials with air flow led a significant increase in n-propanol and isobutanol in comparison with the other fermentation trials, while amylic alcohol was comparable with *S. cerevisiae* pure culture. On the contrary, the wine obtained with *S. bombicola*/*S. cerevisiae* sequential fermentation in aerobic condition, showed a lower amount of β-Phenyl ethanol (rose aroma) than the other wines.

The behaviour of sequential fermentations was variable in the group of esters compounds. Indeed, it was not possible to define a general trend. Indeed, sequential fermentation with 20 mL/L/min of air flow exhibited a significant increase in ethyl butyrate content than other trials and a comparable amount of ethyl hexanoate with other fermentations.

*S. bombicola*/*S. cerevisiae* sequential fermentation in static condition led an increase in isoamyl acetate (banana aroma) content, and a significant decrease of this aroma compound in aerobic condition if compared with *S. cerevisiae* control trial.

*S. bombicola*/*S. cerevisiae* sequential fermentation with air flow affected the acetaldehyde content in comparison with other fermentation trials without negative influence on the aromatic profile of wines. In relation to the main mono-terpens (linalool and geraniol), the resulting wines did not show a significant difference.

## 4. Discussion

In recent years, one of the most relevant concerns that is related to the winemaking sector is the progressive increase of ethanol content. Among microbiological strategies proposed to decrease alcohol level in wine is the use of non-*Saccharomyces* yeasts in co-fermentation or sequential fermentation with *S. cerevisiae* starter strains under aerobic and anaerobic conditions were proposed [24,28,29,31,32,43,44,45,46,47,48]. Several studies reported that the use of air flow during the early stage of fermentation affects yeast physiology and metabolism favouring the fermentation performance of yeasts [49,50,51,52,53]. In particular, in *S. cerevisiae* respiration is repressed by high concentrations of sugars even in the presence of oxygen, whereas in general non-*Saccharomyces* wine yeasts are able to aerobically respire sugar, modulating the production of ethanol, glycerol and other by-products [28,47,54,55,56].

In this work, the effect of aeration on ethanol content, population dynamics and analytical profile of wines using free cells of *S. bombicola*/*S. cerevisiae* sequential inoculation were evaluated. *S. bombicola* strain, used in this work was investigated in a previous work in immobilized form and in anaerobic condition [32], determining an ethanol reduction of 1.6% (*v*/*v*) using 10% (*w*/*w*) of beads corresponding to an inoculation level of 10^8^ cells/mL. Here, a comparable result was obtained (1.46% *v*/*v*) but with a lower inoculum of free cells (5 × 10^7^ cells/mL) and in presence of initial concentration of 10^4^ cells/mL of WNS. Free cell inoculation is an easily procedure to apply at industrial level in winemaking sector in comparison to the use of immobilized cells that in the other side allows high inoculum level.

The ethanol reduction achieved in the present work could be, at least in part, explained by the relevant increase in glycerol as previously reported [38]. A similar result was obtained with *C. zemplinina* (synonym *Starmerella bacillaris*, a closely related species with similar oenological features of *S. bombicola*)*,* that was widely investigated to produce wines with less ethanol levels and higher glycerol content [26].

On the other hand, these results confirmed that the oxygen addition decreased the ethanol production of *S. bombicola* cells highlighting an increase of growth and sugar utilization kinetics. However, different metabolic behaviour of various non-*Saccharomyces* species was exhibited with oxygen supplied, highlighting that it is not possible to delineate a general trend within non-*Saccharomyces* yeasts [57].

*S. bombicola* in sequential fermentation confirmed the highest production of glycerol and succinic acid as previously reported [38]. Moreover, the results showed a positive role of oxygen on cell growth and development of *S. bombicola*. On the other hand, this significant enhancement of by-products together with respiration activity do not completely justify the ethanol reduction obtained and other fermentation products that were not evaluated in this investigation need to be explored.

One of the most negative features in mixed or sequential fermentation non-*Saccharomyces*/*S. cerevisiae* yeasts in aeration condition is the increase of acetic acid, compound responsible of sour and bitter taste [28,48,52,58]. In this study, *S. bombicola* in sequential fermentation both in anaerobic and aerobic condition limiting the air flow in the first 72 h (before the inoculum of *S. cerevisiae*) showed an acetic acid content very closed to that exhibited by *S. cerevisiae* indicating a positive interaction between the two yeast strains.

Conversely, ethyl butyrate and higher alcohols increased with oxygen supplementation. This trend could be related to the oxygen supplementation. Indeed, Valero et al. [59] and Shekhawat et al. [52] showed an increase in the concentration of esters and higher alcohols in aeration condition. The supplementation of oxygen revealed a correlation between higher alcohols content, the growth of non-*Saccharomyces* yeasts, and oxygen levels. However, it is not possible to define the general effect of oxygen on the volatile profile of the wine. Indeed, different factors, such as yeast strains, fermentation conditions and grape variety may concurrently affect the aroma composition of wines [31,60,61].

In conclusion, the results obtained highlighted the ability of *S. bombicola* strain DiSVA 66, in sequential fermentation and under partial aeration conditions, to make wines with reduced alcohol content, thereby maintaining, at the same time, an effective analytical profile. Obviously, it is necessary to set up the modalities of its use in the function of the physiological and fermentation characteristics of the non-*Saccharomyces* specie/strain.

## Figures and Tables

**Table 1:** Main parameters of NGJ fermentation trials. The initial sugar concentration was 218 g/L. Data are means ± standard deviations from three independent experiments. Data with different superscript letters (^a,b,c^) within each Column are different according to Duncan tests (0.05%).

Fermentation Trials	Sugar Consumed (g/L)	Ethanol (% *v*/*v*)	Ethanol Yield (g/g) %	Glycerol (g/L)	Volatile Acidity (as Acetic Acid g/L)	Succinic Acid (g/L)
*S. cerevisiae* pure culture	218 ± 0.00 ^a^	12.12 ± 0.11 ^a^	44.03 ± 0.46 ^a^	3.08 ± 0.27 ^c^	0.35 ± 0.01 ^a^	0.25 ± 0.21 ^b^
*S. bombicola*/*S. cerevisiae* static condition	216.44 ± 0.47 ^a,b^	11.91 ± 0.11 ^b^	43.45 ± 1.13 ^a^	7.30 ± 0.12 ^b^	0.29 ± 0.02 ^b^	0.61 ± 0.14 ^b^
*S. bombicola*/*S. cerevisiae* 20 mL/L/min oxygen	215.03 ± 0.99 ^b^	10.66 ± 0.08 ^c^	38.99 ± 0.73 ^b^	10.50 ± 0.12 ^a^	0.29 ± 0.00 ^b^	2.69 ± 0.10 ^a^

**Table 2:** The main volatile compounds of *S. cerevisiae* pure culture and sequential fermentations with or without air flow addition. Data are means ± standard deviations from three independent experiments. Data with different superscript letters (^a,b,c^) within each Column are different according to Duncan tests (0.05%).

mg/L		Fermentation Trials	
**ESTERS**	*S. cerevisiae* Pure Culture	*S. bombicola*/*S. cerevisiae* 20 mL/L/min	*S. bombicola*/*S. cerevisiae* semi anaerobic condition
Ethyl butyrate	0.41 ± 0.02 ^a,b^	1.08 ± 0.35 ^a^	0.40 ± 0.39 ^b^
Ethyl acetate	30.58 ± 1.27 ^a^	26.17 ± 2.51 ^b^	21.58 ± 1.04 ^c^
Ethyl hexanoate	0.06 ± 0.004 ^a^	0.04 ± 0.011 ^a^	0.03 ± 0.019 ^a^
Isoamyl acetate	2.017 ± 0.05 ^a,b^	0.91 ± 0.34 ^b^	2.71 ± 1.18 ^a^
**ALCOHOLS**			
n-propanol	34.00 ± 2.04 ^b^	69.63 ± 0.06 ^a^	33.74 ± 0.31 ^b^
Isobutanol	14.33 ± 0.16 ^c^	35.34 ± 1.21 ^a^	19.43 ± 2.04 ^b^
Amyl alcohol	4.89 ± 1.77 ^a^	3.82 ± 0.28 ^a^	1.30 ± 0.24 ^b^
Isoamyl alcohol	64.50 ± 2.63 ^a^	45.47 ± 1.36 ^b^	29.31 ± 0.42 ^c^
β-Phenyl ethanol	30.1 ± 0.018 ^a,b^	24.8 ± 0.28 ^b^	35.8 ± 0.07 ^a^
**CARBONYL** **COMPOUNDS**			
Acetaldehyde	10.59 ± 0.19 ^b^	30.12 ± 2.22 ^a^	9.26 ± 0.53 ^b^
**MONOTERPENS**			
Linalool	0.08 ± 0.01 ^a^	0.05 ± 0.001 ^a^	0.12 ± 0.05 ^a^
Geraniol	0.09 ± 0.018 ^a,b^	0.007 ± 0.0004 ^b^	0.10 ± 0.05 ^a^
